# Supplementary material for: Modeling glioblastoma heterogeneity as a dynamic network of cell states
Source: Mol Syst Biol. 2021 Sep 16;17(9):e10105. doi: 10.15252/msb.202010105 (PMC8444284; doi:10.15252/msb.202010105)
Supplement: Supplementary file 5 — Source Data for Figure 3 [file MSB-17-e10105-s001.zip › Figure3A_sourcedata/GSEA_3065/nosigdb_state5.GseaPreranked.1622718435914/ALLEN_111_ASTROL1_6FGFR3ETNPPL.html]

Details for gene set ALLEN\_111\_ASTROL1\_6FGFR3ETNPPL[GSEA]

|  || Dataset | state5 |
| Phenotype | NoPhenotypeAvailable |
| Upregulated in class | na\_pos |
| GeneSet | ALLEN\_111\_ASTROL1\_6FGFR3ETNPPL |
| Enrichment Score (ES) | 0.6520585 |
| Normalized Enrichment Score (NES) | 1.9247388 |
| Nominal p-value | 0.0 |
| FDR q-value | 0.0026595225 |
| FWER p-Value | 0.019 |
Table: GSEA Results Summary

  

Fig 1: Enrichment plot: ALLEN\_111\_ASTROL1\_6FGFR3ETNPPL      
 Profile of the Running ES Score & Positions of GeneSet Members on the Rank Ordered List

  

| PROBE | GENE SYMBOL | GENE\_TITLE | RANK IN GENE LIST | RANK METRIC SCORE | RUNNING ES | CORE ENRICHMENT || 1 | PTN |  |  | 19 | 0.824 | 0.1467 | Yes |
| 2 | ADGRV1 |  |  | 40 | 0.703 | 0.2715 | Yes |
| 3 | SLC1A3 |  |  | 82 | 0.550 | 0.3662 | Yes |
| 4 | PON2 |  |  | 84 | 0.546 | 0.4648 | Yes |
| 5 | MAML2 |  |  | 220 | 0.425 | 0.5264 | Yes |
| 6 | GLIS3 |  |  | 379 | 0.349 | 0.5718 | Yes |
| 7 | FGFR3 |  |  | 567 | 0.293 | 0.6038 | Yes |
| 8 | SLC4A4 |  |  | 598 | 0.286 | 0.6521 | Yes |
| 9 | CST3 |  |  | 1602 | 0.141 | 0.5656 | No |
| 10 | COL5A3 |  |  | 1651 | 0.138 | 0.5851 | No |
| 11 | ZFHX4 |  |  | 1700 | 0.133 | 0.6038 | No |
| 12 | NKAIN3 |  |  | 1766 | 0.129 | 0.6198 | No |
| 13 | NCKAP5 |  |  | 1790 | 0.127 | 0.6403 | No |
| 14 | MEIS2 |  |  | 2207 | 0.098 | 0.6115 | No |
| 15 | KANK1 |  |  | 2429 | 0.083 | 0.6019 | No |
| 16 | SLC7A11 |  |  | 2577 | 0.075 | 0.5990 | No |
| 17 | FGFR2 |  |  | 2817 | 0.062 | 0.5835 | No |
| 18 | GLI3 |  |  | 4538 | -0.016 | 0.3944 | No |
| 19 | NHSL1 |  |  | 5103 | -0.037 | 0.3382 | No |
| 20 | FBXL7 |  |  | 5313 | -0.045 | 0.3229 | No |
| 21 | SHROOM3 |  |  | 6397 | -0.095 | 0.2192 | No |
| 22 | RGS20 |  |  | 7387 | -0.180 | 0.1414 | No |
| 23 | GLUL |  |  | 7594 | -0.203 | 0.1551 | No |
Table: GSEA details [plain text format]

  

Fig 2: ALLEN\_111\_ASTROL1\_6FGFR3ETNPPL: Random ES distribution      
 Gene set null distribution of ES for **ALLEN\_111\_ASTROL1\_6FGFR3ETNPPL**
